# Psychometric Evaluation of the Food Life Questionnaire—Short Form among Brazilian Adult Women

**DOI:** 10.3390/nu16070927

**Published:** 2024-03-23

**Authors:** Edilene Márcia de Sousa, Thainá Richelli Oliveira Resende, Marle dos Santos Alvarenga, António Raposo, Edite Teixeira-Lemos, Raquel Guiné, Hmidan A. Alturki, Pedro Henrique Berbert de Carvalho

**Affiliations:** 1 Body Image and Eating Disorders Research Group (NICTA), Federal University of Juiz de Fora, Governador Valadares 35010-180, MG, Brazil; edilenemarcia@yahoo.com.br (E.M.d.S.); thaina.richelli@gmail.com (T.R.O.R.); 2 Nutrition Department, School of Public Health University of São Paulo, São Paulo 01246-904, SP, Brazil; marlealvarenga@gmail.com; 3 CBIOS (Research Center for Biosciences and Health Technologies), Universidade Lusófona de Humanidades e Tecnologias, Campo Grande 376, 1749-024 Lisboa, Portugal; 4 CERNAS Research Centre, Polytechnic University of Viseu, 3504-510 Viseu, Portugal; etlemos3@gmail.com (E.T.-L.); raquelguine@esav.ipv.pt (R.G.); 5 King Abdulaziz City for Science & Technology, Wellness and Preventive Medicine Institute—Health Sector, Riyadh 11442, Saudi Arabia; halturki@kacst.edu.sa; 6 Eating Disorders Program, Institute of Psychiatry (AMBULIM), University of São Paulo, São Paulo 05403-010, SP, Brazil

**Keywords:** food attitudes, eating attitudes, feeding behaviors, weight concerns, diet–health linkages, validity, reliability, psychometrics, cross-cultural adaptation, measurement

## Abstract

Measures of beliefs and attitudes toward food have generally been limited to the measurement of more pathological eating attitudes (e.g., disordered eating). The Food Life Questionnaire (FLQ) and its short form (FLQ-SF) were developed to examine attitudes toward a broader range of foods; however, the factor structure of the FLQ-SF was not confirmed in any study with young women. In the present study, we performed a psychometric evaluation of the Brazilian Portuguese translation of the FLQ-SF in a sample of 604 women. We evaluated the factor structure using a two-step, split-sample exploratory and confirmatory factor analytic approach. Results supported a four-factor structure (i.e., weight concern, diet–health orientation, belief in a diet–health linkage, and food and pleasure) with 18 items (χ^2^/*df* = 2.09; CFI = 0.95; TLI = 0.94; RMSEA = 0.05 (90% CI = 0.04; 0.06; *p* > 0.05); and SRMR = 0.08). Additionally, we found good internal consistency for all FLQ-SF subscales (McDonald’s *ω* = 0.79–0.89) and convergent validity with measures of feelings, beliefs, and behaviors involved in food attitudes. Collectively, these results support the use of the FLQ-SF in Brazilian women and provide a foundation to expand the literature on beliefs and attitudes toward food in this population.

## 1. Introduction

Food attitudes constitute a comprehensive construct encompassing positive and negative emotions and cognitive and sensory qualities, both general and individual-specific, regarding food [1]. This construct consists of three main components: the affective, the volitional, and the cognitive. The affective component refers to emotions and feelings attributed to sensations and experiences with food [1]. The volitional component is related to will or behavioral intention, where a higher intention to adopt a behavior increases the likelihood of its execution [2,3]. The cognitive component is anchored in beliefs and knowledge acquired throughout life, forming ideas, thoughts, and convictions [2]. Additionally, food attitudes are shaped by environmental factors such as culture, family, and society [4,5].

Rozin et al. [4] found significant cross-cultural differences in food attitudes in a study conducted in four countries (i.e., USA, Japan, Flemish Belgium, and France). These differences are notable, especially in how food functions in the minds and lives of people. While, for some, it serves as pleasure, for others, it represents a stressor and a source of concern, potentially influencing the health of many individuals [4,5]. Such cultural disparities act as determinants in food attitudes [6], revealing distinct cultural values and the complex relationship of these values with food, potentially even reflecting the prevalence of certain chronic diseases, such as cardiovascular diseases [4,5]. On the other hand, research indicates that individuals who adopt a more positive relationship with food can make better dietary choices, assume more balanced eating habits, and have a better perception of their health status [7,8]. A beneficial relationship between individuals, their eating habits, and their bodies reduces the potential risks of psychopathologies related to eating behavior, including eating disorders [9].

To comprehensively assess positive attitudes toward food, some instruments were developed, including the Food Life Questionnaire (FLQ) [4], later refined [5], and its shortened version: Food Life Questionnaire—Short Form (FLQ-SF) [6]. These tools can provide valuable insights into understanding behavioral patterns related to eating.

Initially, the FLQ was developed in four countries (i.e., USA, Japan, France, and Flemish Belgium) [4] to examine cross-cultural differences in food attitudes, evaluate attitudes toward a range of foods, and explore the role of food in daily life. The FLQ measures seven factors: (1) consumption of “healthy” foods (low fat/reduced salt); (2) concern about the healthiness of one’s own and others’ eating habits; (3) the extent of concern about the fattening effects of foods versus savoring food; (4) the importance of food for health; (5) the perception of food as important and pleasurable; (6) nutrition versus culinary associations with food; and (7) self-perception as a healthy eater [4]. The validation study of the FLQ included a sample of university students (*n* = 772) and adults (*n* = 509) of both genders, with an average age ranging between 18 and 37 years. Statistical treatment was carried out through principal component analysis (PCA, with orthogonal Varimax rotation), resulting in 25 items, using the complete sample, resolved into the seven factors mentioned earlier, with eigenvalues greater than 1 [4].

In a later study [5], the scale was refined with American adult university students (*n* = 2162; *M*_age_ = 19.3 years) of both genders (59% women), predominantly White (72%). This study proposed a six-factor structure to replace the original seven factors [3]. These factors include (1) concern about weight; (2) food and health orientation; (3) belief in a diet–health relationship; (4) food negativity/importance; (5) disordered eating characteristics; and (6) natural/vegetarian food preference [5]. PCA was conducted with orthogonal Varimax rotation, similar to the study by Rozin et al. [4]. A six-factor solution was forced (representing 35% of the total explained variance), where only items with factor loadings above 0.40 were considered suitable [5].

Despite its notable importance [4,5], especially in the realm of cultural influences on attitudes toward food, the FLQ faced challenges in terms of applicability, namely a lengthy questionnaire requiring participants to respond to a large number of items and merging response formats (i.e., true/false, Likert scale, and choice between two alternatives) [6]. These limitations prompted Sharp et al. [6] to develop a shortened version (i.e., FLQ-SF), selecting 22 of the 54 items that presented the highest factor loadings reported by Rozin et al. [4]. The short version retains five of the original six factors, namely (1) weight concern; (2) food and health orientation; (3) belief in the diet-health relationship; (4) food and pleasure; and (5) preference for natural/vegetarian foods [6]. In this version, the response format was standardized. All items were answered using a seven-point Likert scale (1 = strongly disagree to 7 = strongly agree), where a higher score indicates a greater emphasis on the measured factor [6].

Sharp et al.’s [6] version included a sample of men and women, predominantly White (87.1%), with an average age of 31 years (*M* = 31.92, *SD* = 14.66). The exploration of the structure, as in previous versions [4,5], was conducted using PCA with orthogonal Varimax rotation. The final structure of the FLQ-SF demonstrated validity and reliability indicators for 21 items of food attitudes, with factor loadings higher than those of Rozin et al. [5], and one item (i.e., item #22) was excluded due to low factor loading [6]. Subsequently, the FLQ-SF was evaluated for the Persian culture with an exclusive sample of overweight and obese individuals, breast cancer survivors, and Iranian women with an average age of 53 years [10]. Through confirmatory factor analysis (CFA), the authors replicated the five-factor structure. However, unlike Sharp et al. [6], Nejati et al. [10] found sufficient factor loadings for all 22 original items.

While the FLQ-SF has demonstrated itself as a valid and reliable measure for assessing food attitudes, psychometrically, the factorial structure and the number of appropriate items for the FLQ-SF (22 versus 21 items) are still not established. This discrepancy may arise due to the use of different techniques for factor analysis, namely, PCA used by Rozin et al. [4,5] and Sharp et al. [6] versus CFA applied in the validation with Iranian women [10]. Although similar in some aspects, these techniques have different objectives and functions [11]. PCA is based only on the linear correlation of the observed variables and does not differentiate the common variance of the specific variance between the items. In factor analysis (i.e., confirmatory factor analysis [CFA]), only the common variance is considered, since it aims to reveal latent constructs that explain the covariance between items; specific variances (individual portions of items) that do not covary with each other are not considered. Therefore, it is the most suitable technique for validating instruments [11,12].

Furthermore, factor rotation was performed using an orthogonal method [4,5,6] suitable for situations in which the instrument factors do not show correlation or when the correlations in oblique rotation are less than 0.30. This is not true for the FLQ-SF, in which its factors scores show correlation between each other (e.g., “belief in the diet–health relationship” and “food and health orientation subscale”). Oblique rotation allows correlated factors instead of maintaining independence among related factors, as only common variance is considered [11].

Another aspect to be highlighted is that in the studies that evaluated and applied the FLQ or its shortened version, the sample presents diverse characteristics, such as differences in gender, age, ethnicity, and health status [4,5,6]. It is known that depending on the sample studied, the latent traits of the construct may be influenced, as is the case with disordered eating behaviors in women compared with men [13,14,15,16]. Research shows that women present lower intuitive eating [9] and higher scores on disordered eating behaviors measures [13,14,15,16] when compared to men. Furthermore, differences in eating attitudes can be found between the young adult population and the average adult [13,15], those with a healthy health status versus those with health problems [13,16], and ethnical groups [16]. Therefore, it is necessary to evaluate the psychometric properties of the FLQ-SF in different cultures and less heterogeneous samples and apply appropriate psychometric analysis techniques to support the choice of the most suitable factorial structure for the FLQ-SF.

In this context, the cross-cultural adaptation and analysis of the validity and reliability of the FLQ-SF for Brazilian women are essential for the assessment of food attitudes in clinical and epidemiological settings. Although there are some validated instruments in Brazil to assess specific food attitudes [17,18,19,20,21,22,23,24,25,26,27], all these instruments aim to screen individuals susceptible to eating disorders (EDs), assessing disordered eating symptoms and risk factors for EDs. In fact, most research on the relationship between food attitudes and healthy eating behaviors has generally been limited to the evaluation and measurement of more pathological eating attitudes [17]. To the best of our knowledge, there are no valid and reliable measures of eating attitudes available to examine attitudes toward a broader range of foods that capture several dimensions of eating attitudes (e.g., weight concern, diet–health eating orientation, beliefs in a diet–health linkage, eating for pleasure and natural food preferences), among Brazilian women.

Finally, the scarcity of instruments for young Brazilian women that assess, in addition to negative eating behaviors, positive aspects can encourage more studies to help understand food attitudes in this population. If it proves valid and reliable, the FLQ-SF could be a tool for formulating interventions that promote improvements in health and reduce the risks of diseases associated with diet. Therefore, in this study, we aimed (1) to analyze the content validity, construct validity (factorial), and convergent validity of the Brazilian Portuguese version of the FLQ-SF and (2) to analyze the reliability (internal consistency) of the Brazilian Portuguese version of the FLQ-SF when applied to a sample of Brazilian young adult women aged 18 to 35 years. It was hypothesized that the Brazilian Portuguese version of the FLQ-SF would provide evidence of content, construct, and convergent validity, as well as robust reliability, among Brazilian young adult women.

## 2. Materials and Methods

### 2.1. Participants

In this validation study, multiple steps were undertaken to translate, culturally adapt, validate, and assess the reliability of the FLQ-SF among Brazilian young adult women [12,28]. A total of 604 Brazilian young adult women participated in the current study. For conducting EFA and CFA, a 10:1 participant-per-item ratio was used [12,29,30].

The specific inclusion criteria were (1) being a Brazilian citizen, (2) self-identifying as a woman, (3) being aged between 18 and 35 years, and (4) having the ability to read and respond to a questionnaire written in Brazilian Portuguese. Exclusion criteria (i.e., having any physician condition that can directly influence eating attitudes, such as tract intestinal diseases) were adopted.

Participants (*n* = 604) had a median age of 25 years (range 18–35) and a median self-reported body mass index (BMI) of 23.47 kg/m^2^ (range 16.03–46.29). The majority of participants described themselves as White (56.46%, *n* = 341), followed by Brown (34.44%, *n* = 208), Black (7.28%, *n* = 44), and Other (1.82%, *n* = 11). In terms of gender identity, 82.78% (*n* = 500) described themselves as cisgender women, 0.83% (*n* = 5) as non-cisgender women, and 16.39% (*n* = 99) preferred not to respond. For sexual orientation, 85.60% (*n* = 517) reported being heterosexual, 1.99% (*n* = 12) lesbian, and 12.41% (*n* = 69) preferred not to respond. Most participants were of high income (73.51%, *n* = 444), followed by mid (20.86%, *n* = 126) and low (0.5%, *n* = 3) income, and 5.13% (*n* = 31) preferred not to respond.

### 2.2. Procedures

The participants were recruited through advertisements posted on social networks (Instagram^®^, Facebook^®^, and WhatsApp^®^) and virtual communities. In addition, invitations were sent by e-mail to higher education institutions with the aim of requesting support in disseminating the research. Furthermore, for greater reach in disseminating the research, posters with a QR code were placed in health services that provide care for women.

Participants were informed of the anonymity and confidentiality of their responses and signed an informed consent form. All procedures were in accordance with the American Psychological Association (APA) Ethical Principles [31], and ethical approval was obtained from the Federal University of Juiz de Fora, Brazil (approval number 5.869.779).

Participants received a link to complete the study instruments on an online platform (Google Forms^®^) accessible on any smartphone, tablet or computer. This tool can make it easier to recruit participants and collect data reliably online. Initially, participants signed the informed consent form and continued responding to the data collection instruments. It is noteworthy that no financial reward was offered for participating in the present study. To ensure that participants responded only once to the survey protocol, IP addresses (internet protocol) were controlled, not allowing access more than once.

### 2.3. Measures

#### 2.3.1. Demographic Data

Sociodemographic information was based on self-reporting and included (a) age, (b) race/ethnicity (i.e., White, Brown, Black, and “Other”) [32], (c) sex assigned at birth, (d) gender identity, (e) sexual orientation, (f) income [33], (g) body mass, and (f) height. The body mass index (BMI) was calculated using the Quetlet index, whose formula is (BMI (kg/m^2^) = weight (kg)/height (m)^2^) [34].

#### 2.3.2. Food Life Questionnaire—Short Form (FLQ-SF)

The FLQ-SF is a 22-item self-report measure that aims to assess beliefs and attitudes toward food [6]. The FLQ-SF factor structure is composed of five subscales: (1) *weight concern* (WC items #1, 2, 3, 4, 5, and 6; e.g., *I am concerned about being overweight)*, (2) *diet–health orientation* (DHO items #7, 8, 9, 10, and 11; e.g., *I am a healthy eater*), (3) *belief in a diet–health linkage* (DHL items #12, 13, 14, and 15; e.g., *Diet can have a big effect on good health*), (4) *food and pleasure* (FP items #16, 17, 18, and 19; e.g., *Enjoying food is one of the most important pleasures in my life*), and (5) *natural food preferences* (NFP items #20, 21, and 22; e.g., *I think natural, organic foods are better for you than commercially grown/processed foods*). Each item is scored on a 7-point Likert-type scale (1 = completely disagree to 7 = completely agree). The higher the score, the greater the emphasis on the measured factor was [6]. The FLQ-SF in South Australia [6] has been shown to have construct, criterion-related, and incremental validity, as well as good internal consistency. In the present study, we applied the Brazilian Portuguese version of the FLQ-SF (please see the cross-cultural translation and adaptation described below).

#### 2.3.3. Intuitive Eating Scale-2 (IES-2)

The IES-2 is a 23-item self-report instrument designed to assess intuitive eating [18]. The IES-2 is composed of four subscales: (1) *unconditional permission to eat* (UPE; 6 items), (2) *eating for physical rather than emotional reasons* (EPRER; 8 items), (3) *reliance on hunger and satiety cues* (RHSC; 6 items), and (4) *body–food choice congruence* (BFCC; 3 items). The structural model also has a second-order factor named *Intuitive Eating*. All items were rated on a 5-point Likert-type scale (1 = never to 5 = always). Higher scores indicate greater intuitive eating. The Brazilian version of the IES-2 has demonstrated good evidence of factorial, convergent, and discriminant validity, as well as good reliability [18]. In the present study, the internal consistency of the IES-2 total score was good (McDonald’s omega [*ω*] = 0.90; 95% confidence interval [CI] = 0.89–0.92), as well as for its subscales: UPE (*ω* = 0.68; 95 CI% = 0.64–0.72), EPRER (*ω* = 0.89; 95 IC% = 0.88–0.91), RHSC (*ω* = 0.91; 95 IC% = 0.90–0.92), and BFCC (*ω* = 0.91; 95 IC% = 0.90–0.92).

#### 2.3.4. Functionality Appreciation Scale (FAS)

The FAS is a one-dimensional, 7-item, self-report measure developed to assess body functionality appreciation [35]. Each item is scored on a 5-point Likert scale (1 = strongly disagree to 5 = strongly agree). The total score is derived from the average of all items. Higher scores are indicative of greater functionality appreciation. The FAS presents indicators of factorial and convergent validity for young Brazilian women and men, as well as adequate internal consistency [35]. In the present study, the internal consistency of the FAS was good (*ω* = 0.94 [95% CI = 0.93–0.95]).

#### 2.3.5. Three-Factor Eating Questionnaire-18 (TFEQ-18)

The TFEQ-18 is an 18-item self-report measure that aims to assess distinct eating behaviors [36]. The TFEQ-18 is composed of three subscales: (1) *restrictive cognitive* (RC; 6 items), (2) *uncontrolled eating* (UE; nine items), and (3) *emotional eating* (EE; three items). The questionnaire is scored as follows: 17 items are answered on a 4-point Likert-type scale, with distinct descriptions between items (#1 to 3; #7 to 13; #17 to 18) as follows: 1 = definitely true to 4 = definitely false; item #4 (1 = almost never to 4 = almost always); item #5 (1 = unlikely to 4 = very likely); item #14 (1 = only at mealtime to 4 = almost always); item #15 (1 = never to 4 = at least once a week). Item #6 is presented as a direct question, answered on an 8-point scale (1 to 8 points), where 1 signifies no restriction on eating (eating what you want, when you want) and 8 signifies total restriction (constantly limiting food intake and never giving in) [36]. The instrument score can range from 18 to 76, where a higher score indicates a greater susceptibility of the individual to develop dysfunctional eating behaviors, such as cognitive restriction, binge eating, and emotional eating. The Brazilian version of the TFEQ-18 shows indications of construct validity for young Brazilian adult women and men, as well as adequate internal consistency [36]. In the present study, the inter-item correlation was performed to calculate the reliability of the RC (*rho* = 0.21–0.77; *ps* < 0.001), given that it has different scoring scores for the items. The internal consistency of the UE (*ω* = 0.82; 95% CI = 0.80–0.84) and EE subscales (*ω* = 0.90; 95% CI = 0.89–0.92) was good.

#### 2.3.6. Body Appreciation Scale-2 (BAS-2)

The BAS-2 is a 10-item self-report measure developed to assess body appreciation [37]. The scale has a one-dimensional structure and is answered using a 5-point Likert-type scale (1 = never to 5 = always). The total score is obtained by summing up the points and can range from 10 to 50. A higher score indicates a greater body appreciation. In its Brazilian version, evidence of construct validity was identified, as well as adequate internal consistency [37]. In the present study, the internal consistency of the BAS-2 was good (*ω* = 0.96; 95% CI = 0.95–0.96).

#### 2.3.7. Food Preoccupation Questionnaire (FPQ)

The FPQ is a 28-item self-reported measure developed to assess the frequency of thoughts and emotional valence in relation to food [38]. The Brazilian Portuguese version of the FPQ [39] is composed of five subscales: (1) *negative emotional valence* (NEG; 8 items), (2) *positive emotional valence* (POS; 6 items), (3) *neutral emotional valence* (NEU; 3 items), (4) *frequency of thoughts* (THOUG; 3 items), and (4) *frequency of planning meals* (FP; 8 items). Each item is scored on a 5-point Likert scale (1 = completely disagree to 5 = completely agree). The higher the score, the greater the emphasis on the measured factor was. The Brazilian Portuguese version of the FPQ showed good construct validity and internal consistency for all subscales for young adult women [39]. In the present study, we found adequate internal consistencies for the NEG (*ω* = 0.89; 95% CI = 0.87–0.91), POS (*ω* = 0.84; 95% CI = 0.84–0.87), NEU (*ω* = 0. 74; 95% CI = 0.69–0.78), THOUG (*ω* = 0. 83; 95% CI = 0.80–0.86), and FP subscales (*ω* = 0.64; 95% CI = 0.57–0.71).

#### 2.3.8. Preference for Intuition and Deliberation in Eating Decision-Making Scale (E-PID)

The E-PID is a 7-item self-report measure that aims to assess decision-making, whether by intuition or deliberation, in relation to food [40]. E-PID is composed of two subscales: (1) *preference for intuition* (INT; 3 items) and (2) *preference for deliberation* (DEL; 4 items) answered on a 5-point Likert scale (1 = completely disagree to 5 = completely agree). The higher the score, the greater the emphasis on the measured factor was. The Brazilian Portuguese version of the E-PID [41] showed good construct validity and internal consistency for both subscales for young adult women. In the present study, adequate internal consistency for the INT (*ω* = 0.77; 95% CI = 0.74–0.80) and DEL subscales (*ω* = 0.82; 95% CI = 0.80–0.84) was good.

### 2.4. Data Analyses

#### 2.4.1. Descriptive Statistics

The missing data were replaced using the expectation maximization method [42] in IBM SPSS Statistics (Version 25). Thus, the final dataset (*n* = 604) was split into two random samples (EFA, *n* = 289, and CFA, *n* = 315) [12]. A univariate normality test of the data was performed using the Shapiro–Wilk test, as well as the asymmetry (*Sk* < 3) and kurtosis (*Ku* < 7) coefficients. Multivariate normality was investigated using the Mardia coefficient (<5). The data presented a non-normal distribution. The categorical variables were described by relative and absolute frequencies, and the numerical data were described by median (*Md*) and minimum–maximum values. To compare the sociodemographic data between the EFA and CFA samples, the chi-squared test and Mann–Whitney *U* test were used.

#### 2.4.2. Factor Structure

EFA with principal-axis factoring and oblique oblimin rotation were conducted to explore the factor structure of the FLQ-SF. The Kaiser–Meyer–Olkin (KMO > 0.80) and Bartlett’s sphericity test (*p* < 0.05) were performed to identify the suitability of the data for factor analysis. To decide on the number of factors to retain in EFA, we used parallel analysis [12]. The factor loadings (λ) matrix was analyzed to identify the correspondence of the items with their respective factors, in which values of ≥0.40 were considered adequate. Items that loaded on more than one factor with λ ≥ 0.32 were considered cross-loadings and were then excluded [12].

CFA with the weighted least square mean and variance adjusted (WLSMV) were performed to confirm the four-factor structure previously identified (i.e., using the EFA) for the FLQ-SF. The model’s adequacy (for both the EFA and CFA) was evaluated using the chi-squared test weighted by degrees of freedom (χ^2^/*df* < 3), root mean-square error of approximation (RMSEA < 0.08; 90% CI; *p* > 0.05), comparative fit index (CFI; values close to 0.95), Tucker–Lewis index (TLI; values close to 0.95), and standardized root mean-square residual (SRMR < 0.08) [12]. The model adjustment was performed using the Lagrange multipliers (i.e., Modification Indices [MI]) when the score was greater than 11 [43].

#### 2.4.3. Convergent Validity and Internal Consistency

The convergent validity was examined via Spearman’s rank order correlation coefficient (*rho*) among the FLQ-SF and IES-2 (total scores and subscales: UPE, EPRER, RHSC, and BFCC), FAS, TFEQ-18 (subscales: RC, UE, and EE), BAS-2, FPQ (subscales: NEG, POS; NEU, THOUG, and FP), and E-PID (subscales: INT and DEL). Following Cohen’s cut-offs, correlations between 0.10–0.29 were considered small, correlations between 0.30 and 0.49 were considered medium, and correlations above 0.50 were considered large [44]. To estimate the internal consistency of the measures, McDonald’s omega (ω) was used, whose values of 0.70 or higher were considered acceptable internal consistency [45].

All analyses were conducted with JASP (JASP team, University of Amsterdam, Amsterdam, The Netherlands) version 0.18.3.0 [46] and used a significance level of *p* < 0.05.

## 3. Results

### 3.1. Cross-Cultural Adaptation

We followed the guidelines for the cross-cultural adaptation of instruments [12,28]. The first step consisted of contacting the first author of the original FLQ-SF validation study [6], who consented to our study. The translation (two independent translators, experts in English), synthesis (accordance between the two translators), and back-translation (two new independent back-translators) were subsequently performed. Next, the final version of the instrument was evaluated by an expert committee composed of two translators (one native and another naive), five eating attitudes experts (nutritionists and health professionals), and two experts in validation studies (both holding a Ph.D.).

The scale translation was considered easily achievable; however, semantic adjustments (i.e., cross-cultural adaptation) were made to make the instrument more understandable for the target population. Regarding the response instructions of the instrument, there was no agreement on the translations of “strongly” and “neither agree nor disagree.” The word “strongly” was translated as *muito* (very) and *totalmente* (completely), while “neither agree nor disagree” was translated as *não concordo nem discordo* and *nem concordo nem discordo* (both meaning “neither agree nor disagree”). There was also no consensus on the translation of item #9. The word “taste” was translated as *sabor* (flavor) and *gosto* (taste). Again, in items #20 and #21, different translations occurred for the term “I think,” which was sometimes translated as *eu acho* and, in other cases, as *eu penso* (both meaning “I think”). The expert committee chose to adapt the terms to those most commonly used in these types of research. Therefore, the term “strongly” was translated as *totalmente*; “neither agree nor disagree” as *não concordo nem discordo*; “taste” as *sabor*; and “I think” as *eu penso*. In the expert meeting, there was also a discussion about the best translation for the word “being,” which was translated as *estar*. In items #2 and #22, the experts suggested adding gender inflection to the words *culpado* (guilty) and *amigo* (friend). In items #20 and #21, the translation of “commercially grown” was debated, and a consensus was reached to use the word *industrializadas* (industrialized). Regarding the scale title, the experts agreed to keep the original name in English (United States) to facilitate the identification of the measure in cross-cultural studies.

Concerning the operational equivalence of the scale, the committee opted to modify the initial scale instructions so that each response option was numbered from 1 (completely disagree) to 7 (completely agree). Finally, a Content Validity Index (CVI) value of 0.95 was found, indicating high agreement among the experts (*n* = 9) [47]. Participants in the pre-test (*n* = 41) had a median age of 28.10 years (between 19–34 years) and demonstrated a good verbal understanding of the FLQ-SF (*M* > 3) on a zero to five-point Likert-type scale (see Supplementary Materials Table S1).

Regarding race/ethnicity, self-identifications were distributed among White (41.5%), Brown (51.2%), and Black individuals (7.3%). The body mass index (BMI), calculated based on self-reported weight and height, ranged from 18.66 to 41.37 kg/m^2^. No suggestions for modifications to the scale were identified. Thus, the final version of the FLQ-SF for application in Brazilian women was obtained.

### 3.2. Descriptive Statistics

Demographic data related to age, BMI, race/ethnicity, gender identity, sexual orientation, and income for both subsamples (EFA and CFA) are available in Table 1. In the psychometric analysis phase, we had the participation of a total of 604 Brazilian young adult women aged between 18 and 35 years, with a body mass index (BMI) ranging from 16.03 to 48.22 kg/m^2^ (*Md* = 23.43). The majority of participants self-identified as White, cisgender, heterosexual, and high-income. It is noteworthy that, concerning demographic data, no statistically significant differences were observed between the EFA and CFA samples (*ps* > 0.05).

### 3.3. Factorial Structure

The sample adequacy measure KMO was 0.81, and Bartlett’s test of sphericity was significant (χ^2^ [231.000] = 2861.310; *p* < 0.001), both indicating that the items of the FLQ-SF were suitable for EFA. Model fit indices were observed, all indicating acceptable fit: RMSEA = 0.07 (90% CI = 0.06–0.08; *p* > 0.05); SRMR = 0.04; TLI = 0.85; and CFI = 0.90. The parallel analysis revealed that items #9 (*Taste is more important to me than nutrition*.), #10 (*I eat low-fat food on a regular basis*), #11 (*I rarely think about the long-term effects of my diet on health.*), and #22 (*I would rather be friends with someone who eats lots of fruits and vegetables than someone who eats lots of meats*.) did not load on any factor (λ < 0.40). Additionally, item #20 (*I think natural, organic foods are better for you than commercially grown/processed foods*.) loaded (λ = 0.41) on the DHL subscale, and item #21 (*I think natural, organic foods taste better than commercially grown/processed foods*.) loaded satisfactorily (λ = 0.48) on the DHO subscale. Moreover, the screeplot indicates a plateau trend starting from the fourth factor (see Supplementary Materials Figure S1). Taken together, these analyses suggest that a four-factor structure rather than the original five factors of the instrument Food Life Questionnaire—Short Form (FLQ-SF) best fits the data. The factor loadings, eigenvalues, and the total explained variance for the subscales are described in Table 2.

A CFA with the robust diagonally weighted least squares (WLSMV) was conducted with the second half of the sample (*n* = 315), where some values proved challenging for the proposed model, necessitating the use of MI. The results from the CFA indicated good fit indices: χ^2^/*df* = 2.24; CFI = 0.95; TLI = 0.94; RMSEA = 0.05 (90% CI = 0.04; 0.06; *p* > 0.05); and SRMR = 0.07. However, item #8 (*I eat fast food on a regular basis*.) showed low factor loading (λ = 0.20) and was, therefore, removed from the model. The re-specified model of the FLQ-SF showed good fit to the data: χ^2^/*df* = 2.09; CFI = 0.95; TLI = 0.94; RMSEA = 0.05 (90% CI = 0.04; 0.06; *p* > 0.05); and SRMR = 0.08; and all items showed a λ above 0.56 (see Supplementary Materials Table S2).

### 3.4. Convergent Validity and Reliability

As expected, the WC subscale demonstrated negative and large associations with the FP, INT, POS, BFCC, and UPE subscales. Moreover, negative and medium correlations were found between the WC subscale and the BAS-2 and IES-2 total scores, and the EPRER, and RHSC subscales. Furthermore, the WC subscale showed positive and medium correlation with the NEU subscale and positive and small correlations with the DEL and THOUG subscales.

Regarding the DHO subscale, a positive and large correlation was found with the BFCC subscale. Moreover, the DHO subscale exhibited negative and medium correlations with the BAS-2 and IES-2 total scores, while small and positive correlations were found for the DHL, FP, THOUG, EPRER, and RHSC subscales and the FAS total score. Furthermore, negative and small correlations were found with the NEG and NEU subscales.

The DHL subscale exhibited a positive and medium correlation with the FS subscale, and it was small and positive with the BAS-2 and IES-2 total scores and the PLAN and BFCC subscales. Negative and small correlations were found between the DHL subscale and the NEG subscale. Moreover, the FP subscale showed positive and medium correlations with the POS, PLAN, UPE, and RHSC subscales and small and positive correlations with the INT, DEL, EPRER, and BFCC and the IES-2 and BAS-2 total scores. Furthermore, a negative and small correlation was found between the FP subscale, the WC subscale and the FAS total score. All correlations between the instruments are shown in Table 3.

Finally, the FLQ-SF demonstrated adequate internal consistency for all the subscales: WC (*ω* = 0.80; 95% CI = 0.77–0.84), DHL (*ω* = 0.89; 95% CI = 0.87–0.91), FP (*ω* = 0.79; 95% CI = 0.75–0.83), and DHO (*rho* = 0.36; 95% CI = 0.28–0.42, *p* < 0.001).

## 4. Discussion

Food attitudes are a construct that is difficult to define and measure, as they involve emotional, cognitive, and behavioral aspects [1,2,3]. Perhaps part of these complexities is due to the lack of valid and reliable measures used cross-culturally to assess them. In this study, we conducted a cross-cultural adaptation and evaluated the psychometric properties of the FLQ-SF [6] for Brazilian adult women. Results from the EFA and CFA did not confirm the five-factor structure with 21 items [6] or the five-factor structure with 22 items [10]. A reduced solution with four factors and 17 items (excluding items #8, #9, #10, #11, and #22) and reshaping the items to the factors was found. Furthermore, the FLQ-SF showed evidence of construct validity, convergent validity, and good internal consistency.

Regarding the cross-cultural process, the FLQ-SF proved to be easy to translate into Brazilian Portuguese and proved to be understood by the target population. Small semantic and idiomatic adaptations were performed to improve the verbal understanding of the target population. The final version demonstrated adequate conceptual, cultural, semantic, idiomatic, and operational equivalence compared with the original version of the instrument when applied to Brazilian adult women.

Results from EFA and CFA showed a better fit for a four-factor structure with 18 items that explain 48.2% of the total variance. Our factor solution differs from that found by Sharp et al. [6] and Nejati et al. [10]. Such discrepancies can be justified by the use of different analytical methods. On the one hand, Sharp et al. [6] applied PCA with an orthogonal Varimax rotation method, albeit inadequate [11,12], finding a five-factor solution with 21 items that explained 60% of the total variance. On the other hand, Nejati et al. [10] used CFA with robust estimation techniques, finding a five-factor solution with 22 items.

In addition to the differences in the analytical procedures, we must highlight the theoretical and statistical decisions taken by Sharp et al. [6] during the development and testing of the FLQ-SF. In short, the authors decided to keep item #6 of the FLQ-SF, even presenting cross-loading between the WC subscale (λ = 0.54) and the DHO subscale (λ = 0.51). Without justifying it, the authors kept item #6 in the WC subscale. Moreover, item #9 showed adequate factor loadings (λ > 0.30) in both the DHO and NFP subscales. It is worth stressing that no a posteriori analysis (e.g., CFA) was conducted to confirm the belongingness of these items in the assigned factors. In our study, item #6 showed high factor loading in the WC subscale, and item #9 was excluded due to low factor loading.

Regarding the excluded items, item #8 (*I eat fast food regularly*.) showed boundary factor loading in the EFA (λ = 0.40) and did not present sufficient factor loading in the CFA, confirming the need for confirmatory analyses on the robustness of a factorial solution. Similarly to the findings of Sharp et al. [6], in our model, item #22 (*I would prefer to be friends with someone who eats lots of fruits and vegetables than someone who eats lots of meats.*) was eliminated because it did not present a satisfactory factor loading in any of the FLQ-SF subscales.

Other factors that may explain the differences found in the present study are the samples used. We evaluated the psychometric properties of the FLQ-SF in a sample of Brazilian young adult women aged 18–35 years, while Nejati et al. [10] evaluated a very specific sample of overweight and obese individuals, breast cancer survivors, and Iranian women, with a mean age of 53 years. Moreover, Sharp et al. [6] focused on a mixed sample of men and women (students and a general sample) with a wide age range (17 to 88 years). Gender, age, health status, and culture can influence the latent traits of the construct [13,14,15,16]. Thus, women tend to exhibit more negative health attitudes when compared to men. This reminds us of the documented difference between men and women in food attitudes [4]. Additionally, a pattern of less restrictive dietary preferences with advancing age has been observed [4].

We did not replicate previous findings by not identifying support for the NFP subscale. The interpretability of this factor by the Brazilian population may have been the cause of its inapplicability to the new context. In general, in Western countries, including Brazil, changes in dietary habits toward a more natural and/or vegetarian diet are still incipient [48]. Additionally, a natural diet is associated with dietary patterns that emphasize unprocessed foods and those closer to their original form, while a vegetarian diet excludes animal-derived foods [49]. However, there is evidence that interventions focused on animal suffering may exert a more persuasive influence on consumers, leading them to alter their diets, compared with health or environmental reasons, as revealed in a recent systematic review [50]. Thus, people who opt for a more natural diet do so for health concerns and socioeconomic/cultural reasons, while the choice to adopt vegetarian diets includes, in addition to these, ethical concerns about animals and environmental impact, as pointed out in a systematic review conducted by Kwasny et al. [51]. These reasons may explain the fact that item #20 (*I think natural, organic foods are better for you than commercially grown/processed foods.*) loaded on the DHL subscale, and similarly, item #21 (*I think natural, organic foods taste better than commercially grown/processed foods.*) loaded on the DHO subscale.

For convergent validity analyses, previous studies [6,10] used the subscales of the FLQ-SF, complete Food Life Questionnaire (FLQ), Food Choice Questionnaire (FCQ), self-report food consumption scores [6], Theory of Planned Behavior (TPB), and semiquantitative food frequency questionnaire (FFQ) [10], finding significant correlations. In the present study, we sought to expand convergent validity analyses by using various instruments assessing constructs similar to the FLQ-SF. Our results indicate an association of the Brazilian version of the FLQ-SF subscales with almost all constructs evaluated in the convergent analysis for samples of adult women (Table 3), providing evidence of convergent validity.

The correlations demonstrated in the study indicate clear aspects of eating attitudes and their particularities. As is the case with concern about body weight (the WC subscale) and food decision-making by deliberation (the DEL subscale) and meal planning (the FP subscale), since individuals who care about their bodies are more likely to make their eating decisions based on external rules and plan them deliberately [40]. This is a fact that can also be justified by the inverse correlation found between concern about weight and aspects such as pleasure, intuition, positive thinking, unconditional permission, and food congruence. These characteristics are also justified by the positive correlation found by the FP subscale with the POS and INT subscales and the IES-2 and BAS-2 total scores, showing that individuals who have a more positive relationship with food care about the pleasure of eating have a higher frequency of positive thoughts about food, intuitive motivation, unconditional permission to eat, internal trust in the signs of hunger and satiety, physical motivation and not emotional eating, congruence between health and food choice and greater appreciation for the body. Furthermore, seeing food as a means of achieving health, and not just as an instrument for body changes, can influence eating for internal reasons and be a motivator for greater body appreciation, as demonstrated by the positive association between the DHL subscale and BFCC subscale and the IES-2 and BAS-2 total scores. Therefore, individual attitudes, choices, and beliefs regarding food play a crucial role in overall health.

Previous studies confirmed that individuals who adopt unhealthy behaviors, such as guilt, fear, and anxiety related to food or use food as compensation for emotional problems, face serious repercussions on psychological, physical, and social well-being, which can lead to eating disorders [52,53]. This suggests that individuals who cultivate a more positive relationship with food tend to adopt more balanced eating habits and, consequently, have a better perception of their general health [8].

Finally, we found adequate internal consistency for all FLQ-SF subscales, with values comparable to those found in previous validation studies [6,10]. However, unlike the study by Sharp et al. [6] and Nejati et al. [10], the internal consistency of the Brazilian version of the FLQ-SF was assessed by McDonald’s omega (*ω*) coefficient, which is an alternative to Cronbach’s alpha [54,55]. Although Cronbach’s alpha coefficient is often applied, it can underestimate the reliability of the construct and is now considered a lower bound of reliability [55].

The present study has some strengths that are worth stressing: (a) recruitment of a considerable sample of adult women and meeting the literature criteria for the adequate number of subjects for validation studies [12]; (b) use of best practices in the cross-cultural adaptation of instruments [12,28]; and (c) use of robust psychometric analyses for factor analysis [12]. Additionally, to the best of our knowledge, this is the first study to assess dietary attitudes through the FLQ-SF in a sample of adult women in the Brazilian context. Despite these contributions, certain limitations should be noted. First, due to the non-probabilistic sample, the results may not be generalizable to all Brazilian women. Second, evaluations were limited to self-reporting, which may introduce participant social desirability bias. It is noteworthy that previous validation studies of the FLQ-SF [6,10] used the same strategy. Third, sample recruitment was done through social networks (i.e., Facebook^®^, Twitter^®^, WhatsApp^®^, and virtual communities), which may result in sample overrepresentation, potentially limiting generalization. Fourth, we did not assess the discriminant validity and temporal stability of the FLQ-SF, and future studies are suggested to incorporate them into their research. Finally, we did not include Brazilian men. Although many studies have shown higher scores in measures of disordered eating behavior [13,14,15,16] and food craving [19] among women compared with men, recent studies have highlighted the need for studies on eating behavior in men [56,57,58,59,60]. Future studies should evaluate the psychometric properties of the FLQ in men.

## 5. Conclusions

The FLQ-SF has been translated and adapted for the Brazilian population, demonstrating robust psychometric indicators of validity and reliability among adult women. Considering that concerns regarding dietary attitudes have received limited attention in the national context, instruments capable of assessing these outcomes can serve as a catalyst for change in predicting the eating behavior of Brazilian women. This, in turn, allows for the proposition of culturally competent intervention strategies for improvements in public policies and the promotion of individual and collective health in the field of dietary concerns. Furthermore, these findings provide a foundation for expanding the literature on dietary attitudes across diverse populations.

## Figures and Tables

**Table 1:** Descriptive statistics and test of differences between exploratory factor analysis (EFA) and confirmatory factor analysis (CFA) samples on demographic data.

Variables	EFA Sample (*n* = 289)	CFA Sample (*n* = 315)	Test Result ^c^
Age (years) ^a^	26 (18–35)	25 (18–35)	*U* = 43448; *p* = 0.333
BMI (kg/m^2^) ^a^	23.43 (16.03–46.29)	23.50 (16.03–46.29)	*U* = 46259; *p* = 0.729
Race/ethnicity ^b^			
White	171 (59.17%)	170 (53.96%)	χ^2^ (9) = 3.196; *p* = 0.362
Brown	97 (33.56%)	111 (35.24%)	
Black	16 (5.54%)	28 (8.89%)	
Other	5 (1.73%)	6 (1.91%)	
Gender Identity ^b^			
Cisgender	237 (82.01%)	263 (83.49%)	χ^2^ (4) = 0.444; *p* = 0.801
Non-Cisgender	2 (0.69%)	3 (0.95%)	
Prefer not to respond	50 (17.30%)	49 (15.56%)	
Sexual Orientation ^b^			
Heterosexual	247 (85.46%)	270 (85.72%)	χ^2^ (9) = 1.25; *p* = 0.740
Lesbian	4 (1.38%)	8 (2.54%)	
Others	35 (12.12%)	34 (10.79%)	
Prefer not to respond	3 (1.04%)	3 (0.95%)	
Income ^b^			
High	212 (73.35%)	232 (73.65%)	χ^2^ (9) = 1.64; *p* = 0.438
Mid	62 (21.45%)	64 (20.31%)	
Low	0 (0%)	3 (0.95%)	
Prefer not to respond	15 (5.20%)	16 (5.09%)	

Note. EFA = exploratory factor analysis; CFA = confirmatory factor analysis; BMI = body mass index. The categories suggested by the Brazilian Institute of Geography and Statistics (IBGE) [32] were used to classify race/ethnicity: White, Brown, Black, and Other (Yellow and Indigenous); Sexual Orientation = Heterosexual, lesbian, and others (asexual, pansexual, bisexual); ^a^ = Results expressed as median, minimum, and maximum values; ^b^ = Results expressed in absolute and relative frequency; ^c^ = Test result for numerical data (Mann–Whitney test—*U*) or categorical data (chi-squared test—*χ*^2^).

**Table 2:** Descriptive statistics and factor loadings for EFA of the Food Life Questionnaire—Short Form (FLQ-SF) among Brazilian young adult women.

FLQ-SF Items/*Brazilian Portuguese Translation*	*Md* (IQR)	Range	Subscales (λ)			
			WC	DHO	DHL	FP
1. I am concerned about being overweight./*Eu me preocupo em estar acima do peso.*	6 (2)	1–7	**0.648**	−0.183	0.106	0.005
2. I feel guilty when I overeat./*Eu me sinto culpado(a) quando como em excesso.*	6 (3)	1–7	**0.739**	−0.246	0.050	−0.026
3. My thighs are too fat./*Minhas coxas são muito gordas.*	3 (4)	1–7	**0.537**	−0.262	−0.053	0.055
4. I consciously hold back at meal time, so as not to gain weight./*Eu conscientemente me seguro na hora das refeições para não ganhar peso.*	2 (4)	1–7	**0.721**	0.120	−0.023	0.005
5. I am currently on a diet./*Atualmente, eu estou fazendo dieta.*	2 (4)	1–7	**0.617**	0.277	−0.079	−0.011
6. I control my caloric intake./*Eu controlo meu consumo de calorias.*	2 (4)	1–7	**0.664**	0.359	−0.008	−0.072
7. I am a healthy eater./*Eu como de forma saudável.*	5 (3)	1–7	0.022	**0.704**	0.019	0.147
8. I eat fast food on a regular basis./*Eu como fast food regularmente*.	5 (3)	1–7	−0.054	**0.404**	−0.059	−0.170
9. Taste is more important to me than nutrition./*Para mim, o sabor é mais importante do que a nutrição.*	4 (3)	1–7	−0.086	0.352	0.108	−0.269
10. I eat low-fat food on a regular basis./*Eu como alimentos com baixo teor de gordura regularmente.*	4 (2)	1–7	0.333	0.393	0.122	0.019
11. I rarely think about the long-term effects of my diet on health./*Eu raramente penso sobre os efeitos a longo prazo que minha dieta terá em minha saúde.*	6 (3)	1–7	−0.047	0.389	0.101	−0.140
12. Diet can have a big effect on good health./*A dieta pode ter um grande efeito em uma boa saúde.*	7 (1)	1–7	0.052	−0.029	**0.839**	−0.035
13. Diet can have a big effect on heart disease./*A dieta pode ter um grande efeito em doenças do coração.*	7 (1)	1–7	−0.021	−0.010	**0.908**	−0.005
14. Diet can have a big effect on obesity./*A dieta pode ter um grande efeito na obesidade.*	7 (1)	1–7	0.027	−0.006	**0.903**	0.024
15. Diet can have a big effect on cancer./*A dieta pode ter um grande efeito no câncer.*	6 (2)	1–7	−0.055	0.036	**0.832**	0.008
16. Enjoying food is one of the most important pleasures in my life./*Desfrutar da comida é um dos prazeres mais importantes da minha vida.*	6 (2)	1–7	0.056	−0.141	0.014	**0.705**
17. I have fond memories of family food occasions./*Eu tenho boas lembranças de família em ocasiões envolvendo comida.*	6 (2)	1–7	0.048	0.073	0.035	**0.725**
18. Money spent on food is well spent./*Dinheiro gasto em comida é um dinheiro bem gasto.*	6 (2)	1–7	0.054	−0.038	0.042	**0.781**
19. I think about food in a positive way./*Eu penso em comida de uma forma positiva.*	6 (2)	1–7	−0.224	0.163	0.006	**0.705**
20. I think natural, organic foods are better for you than commercially grown/processed foods./*Eu penso que comidas naturais, orgânicas, são melhores para você do que comidas industrializadas/processadas.*	6 (2)	1–7	−0.045	0.230	**0.417**	0.255
21. I think natural, organic foods taste better than commercially grown/processed foods./*Eu penso que comidas naturais, orgânicas, são mais gostosas do que comidas industrializadas/processadas.*	5 (3)	1–7	0.026	**0.482**	0.123	0.215
22. I would rather be friends with someone who eats lots of fruits and vegetables than someone who eats lots of meats./*Eu prefiro ser amigo(a) de alguém que come muitas frutas e vegetais do que alguém que come muitas carnes.*	3 (3)	1–7	0.147	0.122	0.153	−0.059
Explained variance percentages (subscales)			**12.8**	**8.5**	**15.6**	**11.3**
Total explained variance			**48.2%**			

Note: *n* = 289; EFA = exploratory factor analysis; FLQ-SF = Food Life Questionnaire—Short Form; IQR = interquartile range; WC = weight concern subscale; DHO = diet–health orientation subscale; DHL = diet–health link subscale; FP = pleasure and food subscale; λ = factorial loading. Values in bold indicate that an item is loaded on the corresponding factor.

**Table 3:** Descriptive statistics and bivariate correlations between the FLQ-SF and convergent measures.

Variables	*Md* (IQR)	Range	FLQ-WC	FLQ-DHO	FLQ-DHL	FLQ-FP
FLQ-WC	23 (13)	6–40	—	0.03	0.01	−0.22 ***
FLQ-DHO	14 (6)	3–21	0.03	—	0.26 ***	0.20 ***
FLQ-DHL	32 (7)	5–35	0.01	0.26 ***	—	0.35 ***
FLQ-FP	23 (6)	4–28	−0.22 ***	0.20 ***	0.35 ***	—
EPID-INT	10 (5)	3–15	−0.24 ***	0.04	0.02	0.26 ***
EPID-DEL	14(6)	4–20	0.18 ***	0.42 ***	0.26 ***	0.12 **
FPQ –NEG	14 (9)	7–35	0.41 ***	−0.25 ***	−0.10 *	−0.24 ***
FPQ-POS	19 (8)	6–30	−0.11 **	−0.05	−0.01	0.32 ***
FPQ-NEU	9 (2)	4–15	−0.03	−0.08 *	0.03	−0.06
FPQ-THOUG	9 (5)	3–15	0.23 ***	−0.19 ***	0.01	0.02
FPQ-PLAN	11 (4)	3–15	−0.02	0.23 ***	0.17 ***	0.30 ***
IES-UPE	22 (6)	6–30	−0.62 ***	−0.07	0.06	0.32 ***
IES-EPRER	23 (13)	8–40	−0.33 ***	0.28 ***	0.06	0.10 *
IES-RHSC	19 (10)	6–30	−0.37 ***	0.29 ***	0.05	0.30 ***
IES-BFCC	11 (5)	3–15	−0.13 **	0.60 ***	0.11 **	0.17 ***
IES-2	75 (14)	34–115	−0.49 ***	0.35 ***	0.10 *	0.28 ***
TFEQ-18 RC	12 (2)	6–21	−0.19 ***	0.00	0.01	0.01
TFEQ-18 UE	23 (8)	6–40	−0.24 ***	0.24 ***	0.12 **	0.07
TFEQ-18 EE	8 (5)	3–21	−0.27 ***	0.28 ***	0.06	0.04
BAS-2	36 (14)	5–35	−0.37 ***	0.37 ***	0.12 **	0.21 ***
FAS	13 (8)	4–28	0.28 ***	−0.31 ***	−0.24 ***	−0.25 ***

Note: *n* = 315. *Md* = median; IQR = interquartile range; FLQ-SF = Food Life Questionnaire—Short Form; WC = weight concern subscale; DHO = diet–health orientation subscale; DHL = diet–health link subscale; FP = pleasure and food subscale; E-PID = Preference for Intuition and Deliberation in Eating Decision-making Scale; INT = intuition preference subscale; E-PID-DEL = deliberation preference subscale; FPQ = Food Preoccupation Questionnaire; NEG = valence subscale negative; POS = valence subscale positive; NEU = valence subscale neutral; THOUG = frequency of thoughts subscale; FP = frequency of planning subscale; IES-2 = Intuitive Eating Scale-2; UPE = unconditional permission to eating; EPRER = eating for physical rather than emotional reasons; RHSC = reliance on hunger and satiety cues; BFCC = body–food choice congruence; TFEQ-18 = Three-Factor Eating Questionnaire-8; RC = cognitive restrictive behavior subscale; UE = uncontrolled behavior subscale; EE = emotional behavior subscale; BAS-2 = Body Appreciation Scale-2; FAS = Functionality Appreciation Scale. * *p* < 0.05; ** *p* < 0.01; *** *p* < 0.001.

## Data Availability

The data presented in this study are available on request from the corresponding author. The data are not publicly available due to not obtaining consent from respondents to publish the data.

## Supplementary Materials

The following supporting information can be downloaded at https://www.mdpi.com/article/10.3390/nu16070927/s1, Table S1: Evaluation of verbal comprehension and content validity of the Brazilian Portuguese version of the Food Life Questionnaire—Short Form (FLQ-SF). Figure S1: Screeplot derived from parallel analysis of EFA (*n* = 289). Table S2: Confirmatory factor analysis and standardized factor loadings of the re-specified model of the Food Life Questionnaire—Short Form (FLQ-SF).
