# Supplementary material for: Psychometric Evaluation of the Food Life Questionnaire—Short Form among Brazilian Adult Women
Source: Nutrients. 2024 Mar 23;16(7):927. doi: 10.3390/nu16070927 (PMC11013054; doi:10.3390/nu16070927)
Supplement: Supplementary file 1 [file nutrients-16-00927-s001.zip › nutrients-2923071-supplementary.pdf]

Supplementary Materials

Table S1. Evaluation of verbal comprehension and content validity of the Brazilian Portuguese version of the Food Life Questionnaire-Short Form (FLQ-SF).

**FLQ-SF items / *Brazilian Portuguese translation***	**Verbal Comprehension *Md* (Min-Max)**	**CVI**
**1** - I am concerned about being overweight. / *Eu me preocupo em estar acima do peso.*	5 (2-5)	1
**2** - I feel guilty when I overeat. / *Eu me sinto culpado(a) quando como em excesso.*	5 (2-5)	1
**3** - My thighs are too fat. / *Minhas coxas são muito gordas.*	5 (0-5)	1
**4** - I consciously hold back at meal time, so as not to gain weight. / *Eu conscientemente me seguro na hora das refeições para não ganhar peso.*	5 (0-5)	1
**5** - I am currently on a diet. / *Atualmente, eu estou fazendo dieta.*	5 (0-5)	1
**6** - I control my caloric intake. / *Eu controlo meu consumo de calorias.*	5 (0-5)	1
**7** - I am a healthy eater. / *Eu como de forma saudável.*	5 (0-5)	1
**8** - I eat fast food on a regular basis. / *Eu como fast food regularmente.*	5 (0-5)	1
**9** - Taste is more important to me than nutrition. / *Para mim, o sabor é mais importante do que a nutrição.*	5 (0-5)	1
**10** - I eat low-fat food on a regular basis. / *Eu como alimentos com baixo teor de gordura regularmente.*	5 (1-5)	1
**11** - I rarely think about the long-term effects of my diet on health. / *Eu raramente penso sobre os efeitos em longo prazo que minha dieta terá em minha saúde.*	5 (0-5)	1
**12** - Diet can have a big effect on good health. / *A dieta pode ter um grande efeito em uma boa saúde.*	5 (2-5)	1
**13** - Diet can have a big effect on heart disease. / *A dieta pode ter um grande efeito em doenças do coração.*	5 (2-5)	1
**14** - Diet can have a big effect on obesity. / *A dieta pode ter um grande efeito na obesidade.*	5 (2-5)	1
**15** - Diet can have a big effect on cancer. / *A dieta pode ter um grande efeito no câncer.*	5 (1-5)	1
**16** - Enjoying food is one of the most important pleasures in my life. / *Desfrutar da comida é um dos prazeres mais importantes da minha vida.*	5 (1-5)	1
**17** - I have fond memories of family food occasions. / *Eu tenho boas lembranças de família em ocasiões envolvendo comida.*	5 (2-5)	1
**18** - Money spent on food is well spent. / *Dinheiro gasto em comida é um dinheiro bem gasto.*	5 (2-5)	1
**19** - I think about food in a positive way. / *Eu penso em comida de uma forma positiva.*	5 (2-5)	1
**20** - I think natural, organic foods are better for you than commercially grown/processed foods. / *Eu penso que comidas naturais, orgânicas, são melhores para você do que comidas industrializadas/processadas.*	5 (0-5)	1
**21** - I think natural, organic foods taste better than commercially grown/processed foods. / *Eu penso que comidas naturais, orgânicas, são mais gostosas do que comidas industrializadas/processadas.*	5 (0-5)	1
**22** - I would rather be friends with someone who eats lots of fruits and vegetables than someone who eats lots of meats. / *Eu prefiro ser amigo(a) de alguém que come muitas frutas e vegetais do que alguém que come muitas carnes.*	4 (0-5)	0
**Total**	**4 (2-5)**	**0.95**

Note: *Md* = median; Min = minimum; Max = maximum; CVI = Content Validity Index.

Figure S1. Scree plot derived from parallel analysis of EFA (n = 289).

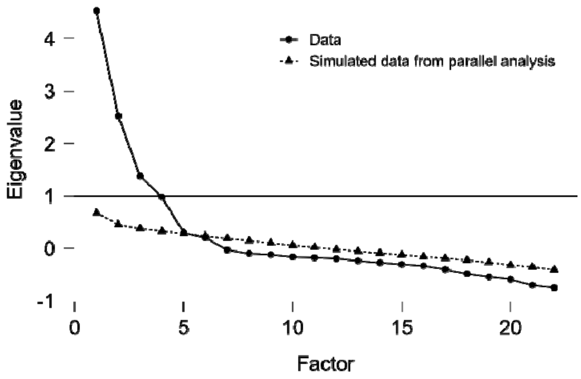

Table S2. Confirmatory factor analysis and standardized factor loadings of the re-specified model of the Food Life Questionnaire-Short Form (FLQ-SF).

						95% CI		
**Subscales**	**Item**	**Non-Standardized λ**	***SE***	**z-value**	***p***	**Lower**	**Upper**	**Standardized λ**
WC	FLQ1	1.250	0.173	7.226	< .001	0.911	1.589	0.648
	FLQ2	1.435	0.183	7.825	< .001	1.075	1.794	0.712
	FLQ3	1.285	0.223	5.758	< .001	0.848	1.722	0.572
	FLQ4	1.228	0.155	7.935	< .001	0.925	1.531	0.661
	FLQ5	0.655	0.048	13.657	< .001	0.561	0.748	0.655
	FLQ6	1.140	0.173	6.600	< .001	0.802	1.479	0.562
DHO	FLQ7	1.159	0.181	6.392	< .001	0.804	1.515	0.650
	FLQ21	1.146	0.190	6.048	< .001	0.775	1.518	0.607
DHL	FLQ12	0.849	0.058	14.663	< .001	0.736	0.963	0.691
	FLQ13	1.196	0.069	17.442	< .001	1.061	1.330	0.867
	FLQ14	1.109	0.059	18.805	< .001	0.993	1.225	0.840
	FLQ15	1.193	0.065	18.497	< .001	1.067	1.320	0.850
	FLQ20	0.785	0.068	11.595	< .001	0.653	0.918	0.603
FP	FLQ16	1.100	0.119	9.226	< .001	0.866	1.333	0.636
	FLQ17	1.103	0.090	12.307	< .001	0.928	1.279	0.743
	FLQ18	0.941	0.082	11.470	< .001	0.780	1.102	0.655
	FLQ19	1.062	0.087	12.170	< .001	0.891	1.233	0.731

Note: n = 315; λ = factor loadings; *SE* = standard error; CI = confidence interval; WC = weight concern subscale; DHO = diet-health orientation subscale; DHL = diet-health link subscale; FP = pleasure and food subscale.
